# Protective effect of miR-33-5p on the M1/M2 polarization of microglia and the underlying mechanism

**DOI:** 10.1080/21655979.2022.2061285

**Published:** 2022-04-29

**Authors:** Song Chai, Yilan Sheng, Ran Sun, Jieshi He, Lihua Chen, Fei He, Wenhua Chen, Dingying Ma, Bo Yu

**Affiliations:** a Department of Rehabilitation Medicine, Shanghai General Hospital, Shanghai Jiaotong University, Shanghai, China; b Department of Rehabilitation, School of International Medical Technology, Shanghai Sanda University, Shanghai, Pudong, China; c Department of Rehabilitation Medicine, Ningbo No. 9 Hospital, Ningbo, Zhejiang Province, China; d Department of Rehabilitation, Shanghai Fifth Rehabilitation Hospital, Shanghai, China

**Keywords:** Microglia, transcript sequencing, M1/M2 polarization

## Abstract

This study was aimed to investigate the influence of miR-33-5p on the M1/M2 polarization of microglia and the underlying mechanism. Transcriptome sequencing was performed using microglia from miR-33-5p mimic and control groups. In total, 507 differentially expressed genes, including 314 upregulated genes and 193 downregulated genes, were identified. The subnetwork of module A, which was extracted from the protein–protein interaction networks, mainly contained the downregulated genes. *Cdk1,Ccnb*,and *Cdc20*, the members of module-A networks with the highest degrees, possess the potential of being biomarkers of ischemic stroke due to their function in the cell cycle. NFY, a transcription factor, was predicted to have the regulatory relation with nine downregulated genes. Overall, our findings will provide a valuable foundation for genetic mechanisms and treatment studies of ischemic stroke.

## Graphic abstract

**Figure:**
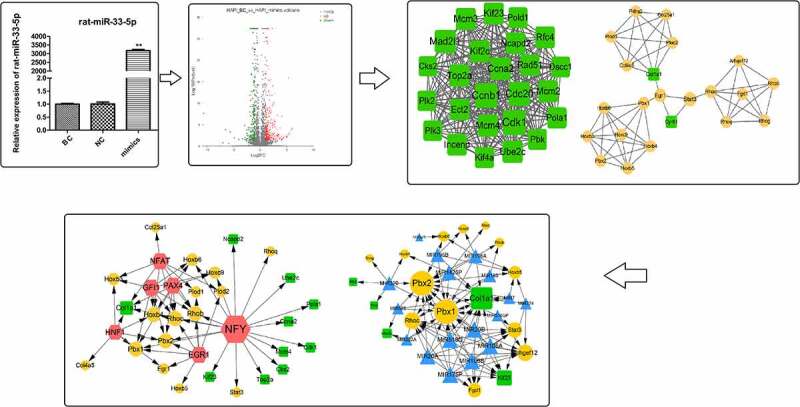

## Highlights

- PCA analysis showed significant differences between HAPI-mimic and blank control groups.
- Cell cycle-related genes, such as Cdk1, Ccnb1, and Cdc20, were identified based on modularized genes.
- The transcription factor NFY regulated nine downregulated genes.

## Introduction

Cardiovascular and cerebrovascular diseases are common and serious threats to humans worldwide [1]. Approximately 80 million people have experienced stroke, and more than 50 million survivors suffer from some form of permanent disability. Cerebral apoplexy is divided into ischemic stroke and hemorrhagic stroke, among which ischemic stroke is the most common [2]. The morbidity, mortality, and recurrence rate of ischemic stroke are extremely high [3]. The pathophysiological basis of ischemic stroke includes cell apoptosis, imbalance in body oxidation and antioxidation, toxicity effects of excitatory amino acids, and cell inflammation [4]. In many neurodegenerative diseases, the inflammatory response is closely related to the activation and polarization of microglia [5], a group of inflammatory cells [6].

Microglia are the smallest cells in the central nervous system, with small nuclei and little cytoplasm [7]. Microglia are mainly concentrated in the telencephalon, basal ganglia, olfactory bulb, and hippocampus, and are the brain’s inherent immune effector cells, participating in dynamic balance and host defense against pathogens and central nervous system diseases [8]. Microglia are activated under pathological conditions, which are named polarization of microglia [9]. Microglial activation is divided into two major phenotypes: classical activation (also known as M1 phenotype) and substitution activation (M2 phenotype) [10]. M1-type microglia is associated with cytotoxicity, superoxide production, and cytokine secretion [11]. The factors released by M1 microglial cells can inhibit tissue repair, destroy the blood-brain barrier, and participate in neuronal degeneration [12]. In contrast to the M1 phenotype, the M2 microglial phenotype exerts anti-inflammatory effects and promotes wound healing and tissue repair. M2-type microglia can also promote the expression of neuroprotective factors and participate in tissue repair and remodeling by changing gene expression [13]. Therefore, it is of great value to inhibit common markers on the surface of M1 microglia to reduce the cytotoxic effect and enhance the beneficial effect of M2 microglia [14]. However, the mechanism of M1/M2 polarization in microglia remains unclear.

MiR-33-5p has been shown to play a crucial role in the inflammatory response [15], macrophage lipid accumulation [16], and cell proliferation [17]. Zeng et al. demonstrated that miR-33-5p may be a potential biomarker for acute ischemic stroke [18]. Direct intracerebral delivery of miR-33 also changed gene expression [19]. Nevertheless, whether miR-33 is associated with the M1/M2 polarization of microglia and thus indirectly participates in the occurrence of ischemic stroke is still unknown.

As a result, this study was aimed to investigate the influence of miR-33-5p on the M1/M2 polarization of microglia and the underlying mechanism. The changes in gene expression after miR-33-5p overexpression were analyzed by RNA sequencing and bioinformatics methods. Western blotting was used to verify the results.

## Materials and methods

### Cell culture and transfection

Rat microglial HAPI cells were purchased from BNCC (Art. No. BNCC340723, Beijing, China). Briefly, HAPI cells were cultured in Roswell Park Memorial Institute (RPMI) 1640 medium with 10% fetal bovine serum at 37°C and 5% CO_2_ in an incubator.

HAPI cells, at ~80% confluence, were harvested using a trypsin detachment solution and inoculated into a 6-well plate at a density of 5 × 10^5^ cells/well. Cells were transfected with miR-33-5p mimics according to the manufacturer’s instructions (GenePharma Co., Ltd, Shanghai, China). After 48 h of transfection, the cell precipitate was collected and lysed with 1 mL TRIzol for qPCR detection.

### Real-time PCR

Real-time PCR was performed as described previously [20]. Briefly, the reverse transcription system contained 4 μL 5× primeScript RT Master MIX (perfect Real Time), 1 μg RNA, and 15 μL RNase Free water (up to 20 μL). RT-PCR was performed using a quantitative PCR (ABI 7500, Thermo Fisher Scientific, MA, USA) in the presence of a fluorescent dye (SYBR Green I; Takara, NJ, USA). The primers used in this study are shown in Table 1.

**Table 1.** The primers used in this study

Primers	Sequencs (5’-3’)
rat-miR-33-5p-F rat-miR-33-5p-R rat-U6-F rat-U6-R β-actin-rat-F β-actin-rat-R rat-CCL2-F rat-CCL2-R rat-IL-1β-F rat-IL-1β-R rat-TNF-α-F rat-TNF-α-R rat-Ym-1-F rat-Ym-1-R rat-CD206-F rat-CD206-R rat-Arg1-F rat-Arg1-R	AGCTCGGTGCATTGTAGTTGC GTGCAGGGTCCGAGGT GCTTCGGCAGCACATATACTAAAAT CGCTTCACGAATTTGCGTGTCAT ATTGCTGACAGGATGCAGAA TAGAGCCACCAATCCACACAG ACCAGCAGCAGGTGTCCCA TGCTTGAGGTGGTTGTGGAA CAGGATGAGGACCCAAGCAC GTCAGACAGCACGAGGCATTT GCCTCTTCTCATTCCTGCTCG TCCGCTTGGTGGTTTGCTAC TGGAGGCTGGAAGTTTGGAT GATGAATGTCTGCCGGTTCTG GTGCCTACTGCCTGCCCTAA TCCCATCGCTCCACTCAAAG GAGAAAGGTCCCGCAGCAT CAGACCGTGGGTTCTTCACAA
rat-Slc7a5-F rat-Slc7a5-R rat-Rhob-F Rat-Rhob-R rat-Smad1-F rat-Smad1-R rat-Rhog-F rat-Rhog-R rat-Mybl2-F rat-Mybl2-R rat-GAPDH-F rat-GAPDH-R	TGGAGCGTCCCATCAAGGT GAGCACGGTCACGGAGAAGA CTCGGCCAAGACCAAGGAG AGCAGTTGATGCAGCCATTCT CAGCGTGTTGGTGGATGGT TCACTGAGGCACTCCGCATA CGCACCGTGAACCTAAACCT GTGGACTGGCAATGGAGAAAC TTGTGGATGAGGATGGGAAGA CCTGGTTGAGCAGGCTGTTAT AGACAGCCGCATCTTCTTGT CTTGCCGTGGGTAGAGTCAT

### Western blotting

After lysis with RIPA lysis buffer, proteins were extracted from the fully lysed sample. Proteins from each sample were separated by sodium dodecyl sulfate (SDS)-polyacrylamide gel electrophoresis and transferred to a PVDF membrane. After transfer, the membranes were incubated with 5% skim milk. Then, the blots were washed thrice with 1× PBS-T (1000 mL 1× PBS + 1 mL Tween-20) for 5–10 min. The primary antibody diluted with 5% skim milk was incubated overnight at 4°C. After washing the membrane six times, secondary antibody was added and transferred to a table concentrator at 37°C for 2 h. Finally, bands were detected using the Millipore ECL system. Tanon Image Software was used for grayscale analysis. P < 0.05 was the screening criterion for significant difference.

### cDNA library construction and transcriptome sequencing

The sequencing experiment was performed using the Illumina TruseqTM RNA sample prep Kit method for library construction. Briefly, total RNA was extracted using TRIzol reagent (Invitrogen) and its concentration and purity were detected using Nanodrop 2000. After reverse transcription, jointing adaptor, and PCR amplification, a cDNA library was constructed. The library was sequenced using an Illumina HiSeq™ 2000 sequencer (Illumina, San Diego, CA, USA).

### Raw reads filtering

To ensure the accuracy of the subsequent analysis, the original sequencing data were filtered by removing joint sequences, low-quality read segments, and high N (N represents uncertain base information) rate sequence. SeqPrep [21] and Sickle [22] were used to remove the joint sequence from reads, sequences of less than 50 bp, and low-quality sequences.

### Mapping and differential expression analysis

Based on the clean data, TopHat2 [23] was used to perform a sequence alignment analysis. Based on the existing reference genome, the mapped reads were assembled and spliced to obtain differentially expressed genes (DEGs) and new transcripts using Cufflinks [24] and StringTie [25]. The screening criteria for DEGs were |log(FC)| > 1 and p-value < 0.05.

### Functional enrichment analysis of differentially expressed genes (DEGs)

The DEGs were subjected to Gene Ontology (biological process; GO BP) and Kyoto Encyclopedia of Genes and Genomes (KEGG) annotation using the common enrichment analysis tool DAVID [26] (version 6.8). The thresholds were count ≥ 2 and p-value < 0.05.

### Construction of a protein–protein interaction (PPI) network of DEGs

The interaction relationship between DEG-coding proteins was predicted and analyzed using the STRING [27] (version 10.0) database (PPI score: 0.15). Cytoscape plugin MCODE (version 1.4.2) was used to analyze the module in the PPI network (score > 5).

Additionally, the module genes were mapped using GO BP and KEGG databases for functional annotation. DAVID [26] (version 6.8) was used to perform the function analyses, with thresholds of count ≥ 2 and p-value < 0.05.

### Transcription Factor (TF)-target and miRNA-target regulatory network prediction

Based on the significant module genes, the Overrepresentation Enrichment Analysis (ORA) method in WebGestalt [28] was used to predict the TF-target and miRNA-target regulatory relation for network construction.

### Statistical analysis

All experiments were repeated three times. Data are shown as mean ± standard deviation. GraphPad Prism 5 (San Diego, CA, USA) was used to analyze the data from this study. One-way analysis of variance was used for comparisons among groups, followed by Newman-Keuls multiple comparison test. Statistical significance was considered for p-values less than 0.05.

## Results

### Expression of miR-33-5p and M1/M2 biomarkers

**Figure 1.**
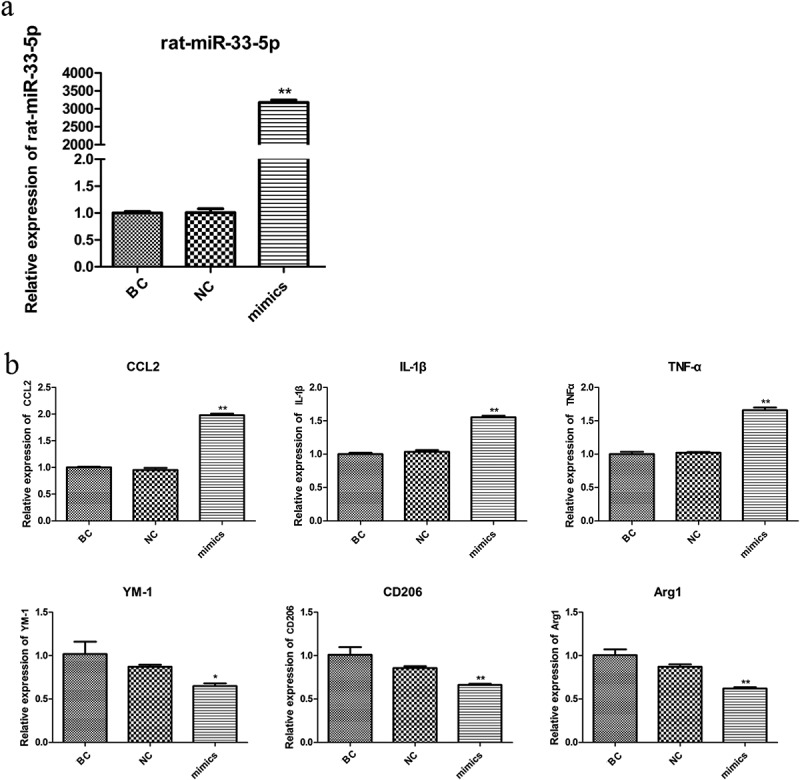
The expression of miR-33-5p (a) and biomarkers of M1/M2 microglia (b). *p < 0.05 and ** p < 0.01.

The expression level of miR-33-5p was detected by RT-PCR. As shown in Figure 1(a), the expression of miR-33-5p in the mimic group was significantly higher than that in the blank control (BC) and negative control (NC) groups (p < 0.01). The biomarkers of M1 microglia (*CCL2, IL-1*, and *TNF-α*) and biomarkers of M2 microglia (*Ym-1, CD206*, and *Arg1*) were detected. The expression levels of the three biomarker genes of M1 in the mimic group were significantly increased compared with those in the BC and NC groups, while M2 in the mimic group were significantly reduced compared with those in the BC and NC groups (Figure 1(b)).

### Genes differentially expressed upon miR-33-5p overexpression

**Figure 2.**
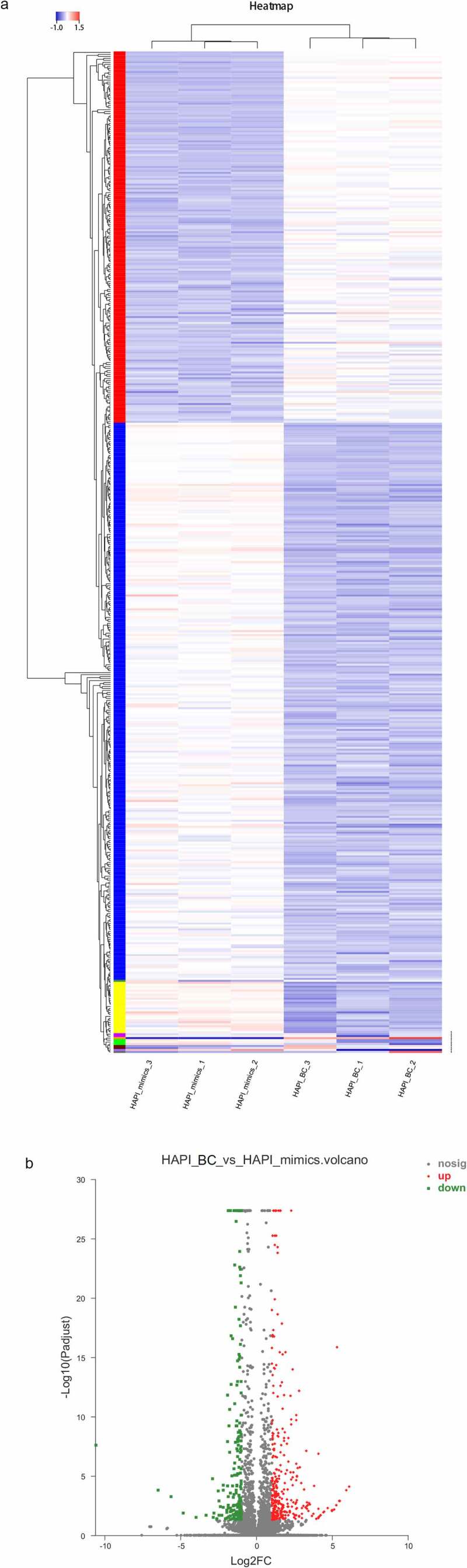
The heatmap (a) and volcano plot (b) of differentially expressed genes.

To investigate the action mechanism of miR-33-5p in the M1/M2 polarization of microglia, DEGs between the groups with or without miR-33-5p mimic treatment were identified. In total, 507 DEGs were found, which included 314 upregulated genes and 193 downregulated genes. The heatmap and volcano plot of DEGs are shown in Figure 2(a-b).

### Functional enrichment analysis of differentially expressed genes (DEGs)

The upregulated DEGs were significantly enriched in 84 BP terms, such as positive regulation of transcription from RNA polymerase II promoter, and regulation of transcription from RNA polymerase II promoter, and 6 KEGG pathways, such as MAPK signaling pathway and transcriptional misregulation in cancer. The downregulated DEGs were significantly enriched in 59 BP terms, such as, mitotic DNA replication initiation, and DNA unwinding involved in DNA replication, and 8 KEGG pathways, such as cell cycle, and DNA replication. The top 5 terms for the enrichment results are shown in Tables 2 and 3.

**Table 2.** Pathways and BP terms (top 5) enriched by upregulated DEGs

Category	Term	Count	PValue
PATHWAY	rno04010:MAPK signaling pathway	12	5.16E-03
PATHWAY	rno05202:Transcriptional misregulation in cancer	9	9.38E-03
PATHWAY	rno00500:Starch and sucrose metabolism	4	1.25E-02
PATHWAY	rno04213:Longevity regulating pathway – multiple species	5	1.63E-02
PATHWAY	rno05135:Yersinia infection	7	1.72E-02
GO_BP	GO:0045944~ positive regulation of transcription from RNA polymerase II promoter	42	7.89E-07
GO_BP	GO:0006357~ regulation of transcription from RNA polymerase II promoter	44	6.59E-06
GO_BP	GO:0048704~ embryonic skeletal system morphogenesis	8	2.44E-05
GO_BP	GO:0007399~ nervous system development	14	4.30E-05
GO_BP	GO:0086010~ membrane depolarization during action potential	5	6.75E-05

**Table 3.** Pathways and BP terms (top 5) enriched by downregulated DEGs

Category	Term	Count	PValue
PATHWAY	rno04110:Cell cycle	11	4.31E-07
PATHWAY	rno03030:DNA replication	6	2.16E-05
PATHWAY	rno04914:Progesterone-mediated oocyte maturation	6	2.11E-03
PATHWAY	rno04114:Oocyte meiosis	6	6.59E-03
PATHWAY	rno03008:Ribosome biogenesis in eukaryotes	5	1.07E-02
GO_BP	GO:1902975~ mitotic DNA replication initiation	4	1.10E-05
GO_BP	GO:0006268~ DNA unwinding involved in DNA replication	5	2.96E-05
GO_BP	GO:0045944~ positive regulation of transcription from RNA polymerase II promoter	26	3.90E-05
GO_BP	GO:0000727~ double-strand break repair via break-induced replication	4	1.16E-04
GO_BP	GO:0045893~ positive regulation of transcription, DNA-templated	17	3.32E-04

### Protein–protein interaction (PPI) network and module analysis

**Figure 3.**
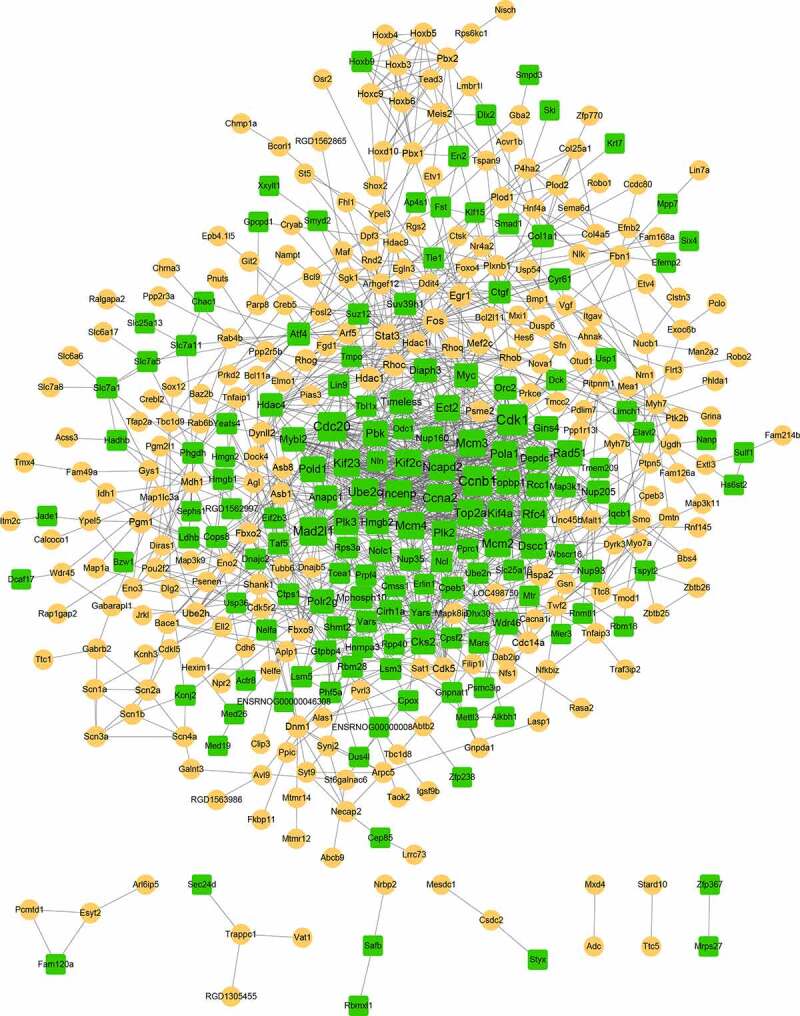
The constructed PPI network. The yellow circle represents upregulated gene, and the green square represents downregulated gene. The size of the node is based on the degree value, with higher degree values indicated by larger nodes.

**Figure 4.**
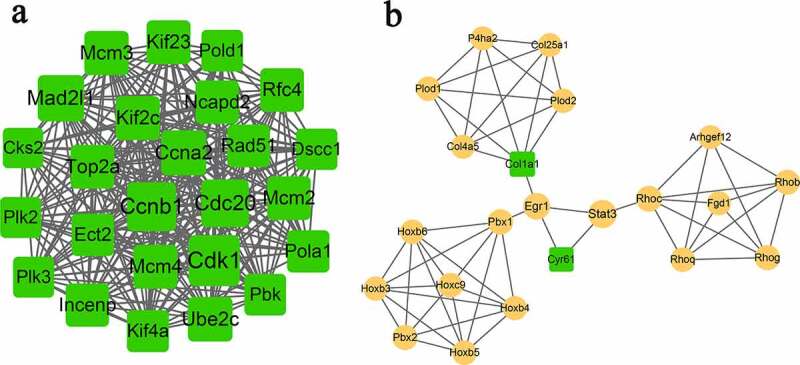
Subnetworks (a, module-A; b, module-B) of PPI network. Yellow circles indicate upregulated genes, and green squares indicate downregulated genes. The size of a node is based on the degree value, with higher degree values indicated by larger nodes.

To obtain more interactions, PPI networks were constructed using STRING. As shown in Figure 3, 407 nodes and 1347 edges were included in the networks. The top ten nodes, with higher degrees, were *Cdk1, Ccnb1, Cdc20, Mad2l1, Ccna2, Ube2c, Mcm3, Mcm4, Kif2c*, and *Kif23*. Due to the large number of nodes in the network, we further selected the key module from the network. Two modules were finally obtained with the threshold of score > 5, as shown in Table 4 and Figure 4. Module A (score: 23.33) contained 25 nodes and 280 edges. All of the genes in module A were downregulated, and the top five were *Cdk1, Ccnb1, Cdc20, Mad2l1*, and *Ccna2*. Module B (score: 5.24) contained 22 nodes and 55 edges (Figure 3). Most genes in this module were upregulated except for *Col1a1* and *Cyr61*.

**Table 4.** Genes in module-A and module-B

module-A			module-B		
Nodes	Description	Degree	Nodes	Description	Degree
Cdk1	down	55	Stat3	up	24
Ccnb1	down	49	Egr1	up	18
Cdc20	down	45	Rhoc	up	14
Mad2l1	down	41	Pbx1	up	12
Ccna2	down	37	Col1a1	down	12
Ube2c	down	36	Pbx2	up	11
Mcm3	down	35	Rhog	up	11
Kif23	down	34	Rhob	up	10
Mcm4	down	34	Hoxc9	up	9
Kif2c	down	34	Plod1	up	8
Top2a	down	33	Rhoq	up	8
Mcm2	down	33	Cyr61	down	8
Rfc4	down	33	Hoxb6	up	8
Incenp	down	32	Hoxb5	up	8
Ncapd2	down	32	Hoxb3	up	7
Rad51	down	30	Plod2	up	7
Ect2	down	29	Hoxb4	up	7
Pola1	down	29	P4ha2	up	6
Kif4a	down	28	Fgd1	up	6
Pold1	down	27	Col4a5	up	6
Pbk	down	27	Arhgef12	up	6
Dscc1	down	26	Col25a1	up	5
Plk3	down	25			
Plk2	down	25			
Cks2	down	23			

### Function analysis of module genes

Genes in module A were significantly enriched in six KEGG pathways, including cell cycle, DNA replication, oocyte meiosis, progesterone-mediated oocyte maturation, and foxo signaling pathway. For GO BP, microtubule-based movement, mitotic cell cycle, cell division, and DNA unwinding involved in DNA replication terms were significantly enriched. The top five BP terms of module B were anterior/posterior pattern specification, embryonic skeletal system morphogenesis, embryonic skeletal system development, positive regulation of transcription from RNA polymerase II promoter, and cellular response to hormone stimulus.

### Transcription factor (TF)-target and miRNA-target networks

**Figure 5.**
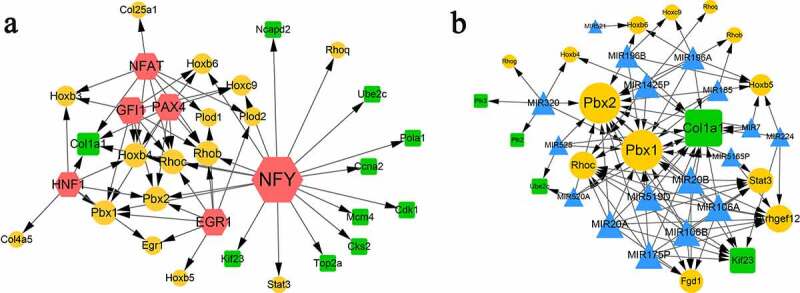
TF-target (a) and miRNA-target (b) networks. Yellow circles indicate upregulated genes. Green squares indicate downregulated genes. Blue triangles indicate predicted miRNAs. Red hexagons indicate transcription factors.

In total, 6 TFs were predicted for the module genes, involving 58 pairs of TF-target regulatory relationships. As shown in Figure 5(a), the six TFs were NFY, NFAT, GFI1, PAX4, HNF1, and GER1. NFY had the highest degree, which regulated the most target genes, such as the downregulated genes of *Ncapd2, Ube2c, Pola1, Ccna2, Cdk1, Mcm4*, etc., and upregulated genes of *Rhoq, Stat3, Pbx2*, etc. GFI1, NFAT, PAX4, and EGR1 regulated eight target genes, respectively. HNF1 regulated seven target genes. The downregulated gene of *Col1a1* was regulated by four TFs, including NFY, NFAT, PAX4, and HNF1.

Based on the module genes, 17 miRNAs were predicted, such as MIR106A, MIR106B, MIR20B, and MIR519D. The miRNA-target network was conducted, which included 17 miRNAs and 18 genes (five downregulated and 13 upregulated), involving 99 regulatory relation pairs (Figure 5(b)). Among the 18 genes, *Pbx1, Pbx2*, and *Col1a1* were the center nodes with degrees greater than 10. In addition, *Arhgef12, Rhoc, Kif23*, and *Stat3* also showed high connectivity degrees with the miRNAs.

### Verification of differentially expressed genes (DEGs) by qPCR and western blotting

**Figure 6.**
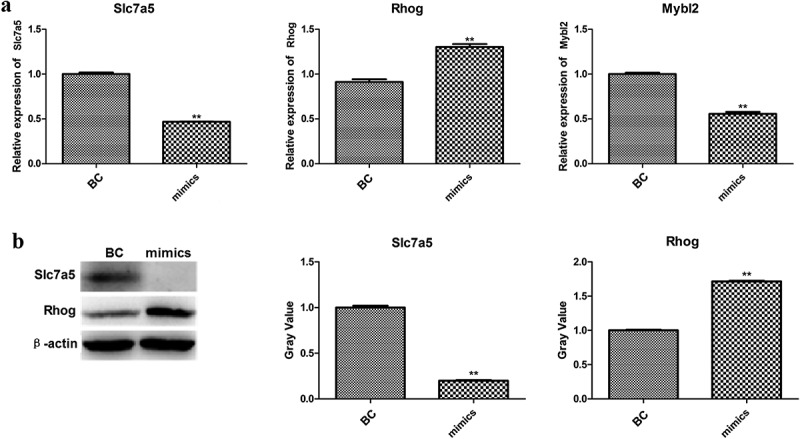
The mRNA (a) and protein levels (b) of verified genes.

Efferocytosis-related genes, *Slc7a5, Rhob, Smad1, Rhog*, and *Mybl2*, were selected from DEGs and their expression levels were verified by qPCR and western blotting. As shown in Figure 6(a), the mRNA expression levels of *Slc7a5, Rhog*, and *Smad1* were significantly different between the two groups. After that, the protein levels of *Slc7a5* and *Rhog* were detected by western blotting. As shown in Figure 6(b), *Slc7a5* was significantly downregulated, while *Rhog* was significantly upregulated in the miR-33-5p mimic group.

## Discussion

In this study, gene expression data were analyzed to identify genes involved in microglia upon overexpression of miR-33-5p. Compared with the control groups, 507 DEGs were identified in groups with mimics. *Cdk1, Ccnb1*, and *Cdc20* had higher degrees in the PPI module. TFs of *NFY, NFAT, GFI1, PAX4, HNF1*, and *GER1* had regulatory relationships with the DEGs.

The differential expression of *Slc7a5* and *Rhog* and the proteins encoded by them was verified by RT-PCR and western blotting, respectively. *Slc7a5* plays a critical role in cell growth and proliferation [29]. To our knowledge, this is the first report to demonstrate the regulatory relationship between miR-33-5p and *Slc7a5*. There is convincing evidence that *Slc7a5* is deeply involved in the occurrence of ischemic stroke [30]. Therefore, more detailed studies are needed to prove the regulatory relationship between *Slc7a5* and miR-33-5p. *Rhog* is a member of the Rho family, which plays an important role in regulating cytoskeletal reorganization in physiological and pathophysiological situations [31]. To our best knowledge, there was no report about the associations between *Rhog* and miR-33-5p or ischemic stroke; therefore, we hypothesized that *Rhog* might participate in M1/M2 polarization based on our results.

All the genes in module A were downregulated. Among the 25 genes in module A, *Cdk1, Ccnb1*, and *Cdc20* possessed the most interactions with other genes. Cyclin-dependent kinases (*Cdks*) have already been reported to mediate the death of ischemic neuronal cells. Zhang et al. proved that the expression of *Cdk1* was induced when primary cortical neuron cultures were exposed to oxygen–glucose deprivation (OGD) for 4 h [32]. *Cdk1* also showed partial resistance to OGD-induced neuronal cell death [33]. Moreover, *Cdk1* has also been shown to play a critical role in neuronal death and has been reported to contribute to the pathogenesis of neurodegenerative diseases [34]. Currently, it is generally accepted that *Cdk1* regulates the cell cycle. Importantly, miR-33 has been demonstrated to play a crucial role in cell proliferation and cell cycle progression by modulating the expression of *Cdk1* [35,36]. Our results were consistent with the abovementioned studies, indicating that the interactions between miR-33 and *Cdk1* may affect the development of ischemic stroke.

Cyclin B1 (*Ccnb1*), an important regulator of the cell cycle machinery, is essential for mouse embryonic development [37]. Several studies have shown that *Ccnb1* is involved in central nervous system regeneration driven by microglia [38]. However, there was no evidence to prove the direct regulation between *Ccnb1* and miR-33-5p. *Cdc20* is an important cell-cycle regulator for the completion of mitosis in organisms [39]. Lloyd et al. found that *Cdc20* could promote the proliferation of microglia through its population replacement process [40]. Elevated *Cdc20* increased extensive mitotic errors, leading to chromosome mis-segregation [41]. Based on the existing literature, we speculated that miR-33-5p may regulate the expression of genes involving in caryomitosis and cell cycle, such as *Cdk1, Ccnb1*, and *Cdc20*.

Interestingly, we found that collagen type Ι alpha Ι (*Col1a1*) appeared in module-B, TF-target, and miRNA-target networks. It has been reported that *Col1a1* is highly related to osteoporotic fracture [42], bone mineral density, and osteoporotic fracture [43]. The only research that associated *Col1a1* with ischemic stroke was completed by Choi et al., who investigated the changes in gene expression after ischemic stroke [44]. In our results, the TF of nuclear factor Y (NFY) showed a wide range of interactions with nine downregulated genes. NFY was proved to be associated with the sterol regulation of human fatty acid synthase promoter I [45]. However, no studies have identified the direct relationship between NFY and microglia or ischemic stroke. We hypothesize that NFY may be involved in microglial polarization by indirectly regulating other genes.

## Conclusions

In conclusion, our result for the first time demonstrated that miR-33-5p plays a crucial role in the M1/M2 polarization of microglia. Overexpression of miR-33-5p induced a significant change in the expression of *Slc7a5* and *Rhog*. Genes that regulate neuron cell cycle and death, such as *Cdk1, Ccnb1*, and *Cdc20*, attracted our attention due to their high potential for M1/M2 polarization.

## Data Availability

The data that support the findings of this study are available from the corresponding author upon reasonable request.
